# miR-223 Inhibits Lipid Deposition and Inflammation by Suppressing Toll-Like Receptor 4 Signaling in Macrophages

**DOI:** 10.3390/ijms161024965

**Published:** 2015-10-20

**Authors:** Jun Wang, Xiaojun Bai, Qiang Song, Fenling Fan, Zhi Hu, Gesheng Cheng, Yushun Zhang

**Affiliations:** Department of Cardiology, the First Affiliated Hospital of Medical College, Xi’an Jiaotong University, Xi’an 710004, China; E-Mails: xiaojunbai@126.com (X.B.); qiangsongxa@163.com (Q.S.); fenlingfan@163.com (F.F.); zhihusx@163.com (Z.H.); geshengcheng@163.com (G.C.)

**Keywords:** atherosclerosis, inflammatory, lipid accumulation, miR-223, PI3K/AKT, TLR4

## Abstract

Atherosclerosis and its complications rank as the leading cause of death with the hallmarks of lipid deposition and inflammatory response. MicroRNAs (miRNAs) have recently garnered increasing interests in cardiovascular disease. In this study, we investigated the function of miR-223 and the underlying mechanism in atherosclerosis. In the atherosclerotic ApoE^−/−^ mice models, an obvious increase of miR-223 was observed in aortic atherosclerotic lesions. In lipopolysaccharide (LPS) activated macrophages, its expression was decreased. The miR-223 overexpression significantly attenuated macrophage foam cell formation, lipid accumulation and pro-inflammatory cytokine production, which were reversed by anti-miR-223 inhibitor transfection. Mechanism assay corroborated that miR-223 negatively regulated the activation of the toll-like receptor 4 (TLR4)-nuclear factor-κB (NF-κB) pathway. Pretreatment with a specific inhibitor of NF-κB (pyrrolidinedithiocarbamate, PDTC) strikingly abrogated miR-223 silence-induced lipid deposition and inflammatory cytokine production. Furthermore, PI3K/AKT was activated by miR-223 up-regulation. Pretreatment with PI3K/AKT inhibitor LY294002 strikingly ameliorated the inhibitory effects of miR-223 on the activation of TLR4 and p65, concomitant with the increase in lipid deposition and inflammatory cytokine production. Together, these data indicate that miR-223 up-regulation might abrogate the development of atherosclerosis by blocking TLR4 signaling through activation of the PI3K/AKT pathway, and provides a promising therapeutic avenue for the treatment of atherosclerosis.

## 1. Introduction

Cardio-cerebral vascular disease, currently the leading cause of mortalities and illness globally, has become the pre-eminent health concern worldwide [1]. Atherosclerosis constitutes the most important contributor to this growing burden of cardio-cerebral vascular complications, including strokes, and myocardial infarction [2,3,4]. Convincing evidence indicates that atherosclerosis is characterized by lipid accumulation and inflammation. Recently, numerous studies have focused on lowering lipid levels and inflammation during atherosclerosis development and vulnerable plaque stabilization, suggesting a potential aspect against cardio-cerebral vascular disease [5,6].

Macrophages rank as the principal effector cells during atherosclerosis. Once activated by various stimulation such as lipopolysaccharide (LPS), macrophages can uptake oxidized low-density lipoprotein (ox-LDL) to induce macrophage foam cell production and the subsequent fatty streak formation, the hallmarks of atherosclerosis [7]. Simultaneously, its activation also has a crucial role in the initiation and propagation of the inflammatory response during atherosclerosis by the production of inflammatory cytokines, such as interleukin-6 (IL-6) and nitric oxide (NO) [8]. Blocking lipid accumulation in foam cells and the consequent inflammatory response are recently understood to be critical for preventing atherosclerotic plaque formation and destabilization. During this process, Toll-like receptors (TLRs) play an important role, especially TLR4 [9,10]. The expression of TLR4 has been shown to be up-regulated in atherosclerotic plaque macrophages. Activation of TLR4 signaling accelerates the uptake of ox-LDL and foam cell formation [11,12]. Its deficiency can reduce intimal lipid deposition and inflammatory cytokines levels in ApoE KO mice, with the subsequent decrease in atherosclerosis plaque [11,13]. Moreover, suppressing the TLR4 pathway by statins elicits plaque stabilization and inflammation reduction in atherosclerotic plaque [14]. Therefore, studies on the mechanisms of negatively regulating TLR4-mediated signaling are pivotal for the cure of atherosclerosis-associated diseases [15].

MicroRNAs (miRNAs) are non-coding small RNA molecules and can inversely regulate their target gene expression at the posttranscriptional level by interacting with the 3ʹ-untranslated region (3ʹ-UTR). Numerous studies have demonstrated that miRNAs are involved in the inflammatory process of cardio-cerebral vascular diseases, such as atrial fibrillation and atherosclerosis [16,17,18]. For example, ectopic expression of miR-155 has been observed in atherosclerotic plaques and proinflammatory macrophages; its deficiency obviously reduces plaque size in ApoE^−/−^ mice [18]. The miR-146 was identified as a potent negative regulator of the TLR4 signaling pathway [19]. miR-147 is induced upon TLR stimulation and negatively regulates murine macrophage inflammatory responses [20]. Besides these, miR-132 [21], miR-21 [22,23] and miR-628 [24] are all involved in TLR signaling. The miR-24, miR-30b, and miR-142-3p were all identified be to be involved in macrophage differentiation [25]. Recently, abundant research has not only confirmed the important function of miR-223 during influenza or hepatitis B infection, leukaemia and breast cancer, but also corroborated its association with several inflammatory diseases, such as inflammatory bowel disease, type 2 diabetes and rheumatoid arthritis [26,27,28,29]. In murine macrophages, the expression of miR-223 was significantly decreased after LPS stimulation [30]. The down-regulated miR-223 promoted the production of pro-inflammatory cytokines triggered by LPS, via signal transducer and activator of transcription 3 (STAT3) [30]. In patients with tuberculosis, miR-223 can regulate macrophage function by inhibition of cytokine production and NF-κB activation [31]. miR-223 also plays a critical role in osteoclast differentiation. miR-223 overexpression can block multinucleated cell formation compared with control cells [32]. miR-223 was also involved in human granulocytic differentiation [33]. Recently, miR-223 was found increased in the aorta of ApoE KO mice [34]. However, its role in atherosclerosis and the underlying mechanism is not fully undefined.

Here, we sought to investigate the expression of miR-223 in atherosclerotic plaque and its effects on lipid accumulation and inflammation responses in LPS-activated macrophages. Furthermore, the potential mechanism was also explored.

## 2. Results

### 2.1. Expression of miR-223 Is Up-Regulated in Atherosclerotic Lesions in ApoE^−/−^ Mice

**Figure 1:**
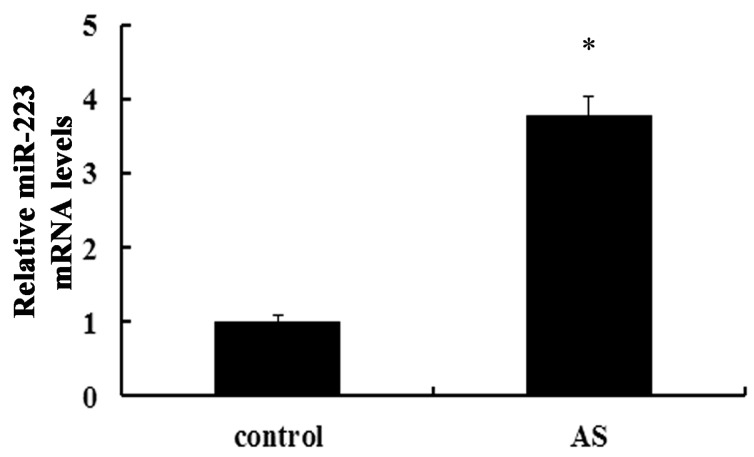
Expression of miR-223 in atherosclerotic lesions. Following construction of atherosclerotic models of ApoE^−/−^ mice by fed with the high-fat diet (HFD) for three months, the expression levels of miR-223 were determined by qRT-PCR assay in atherosclerotic lesion areas. Wild type mice were used in control group. AS: atherosclerosis; * *p* < 0.05 *vs.* control group.

To analyze the expression of miR-223 during atherosclerosis, we quantified its expression in mice models of high-fat diet (HFD)-induced atherosclerotic lesion. As shown in Figure 1, the expression of miR-223 was obviously increased at three months later in HFD-feeding atherosclerotic groups, compared with the wild type control group. Therefore, this result confirmed an increase of miR-223 in atherosclerotic lesion, indicating a potential function of miR-223 in the development of atherosclerosis.

### 2.2. Down-Regulation of miR-223 Was Confirmed in Macrophages Stimulated with TLR Agonists

**Figure 2:**
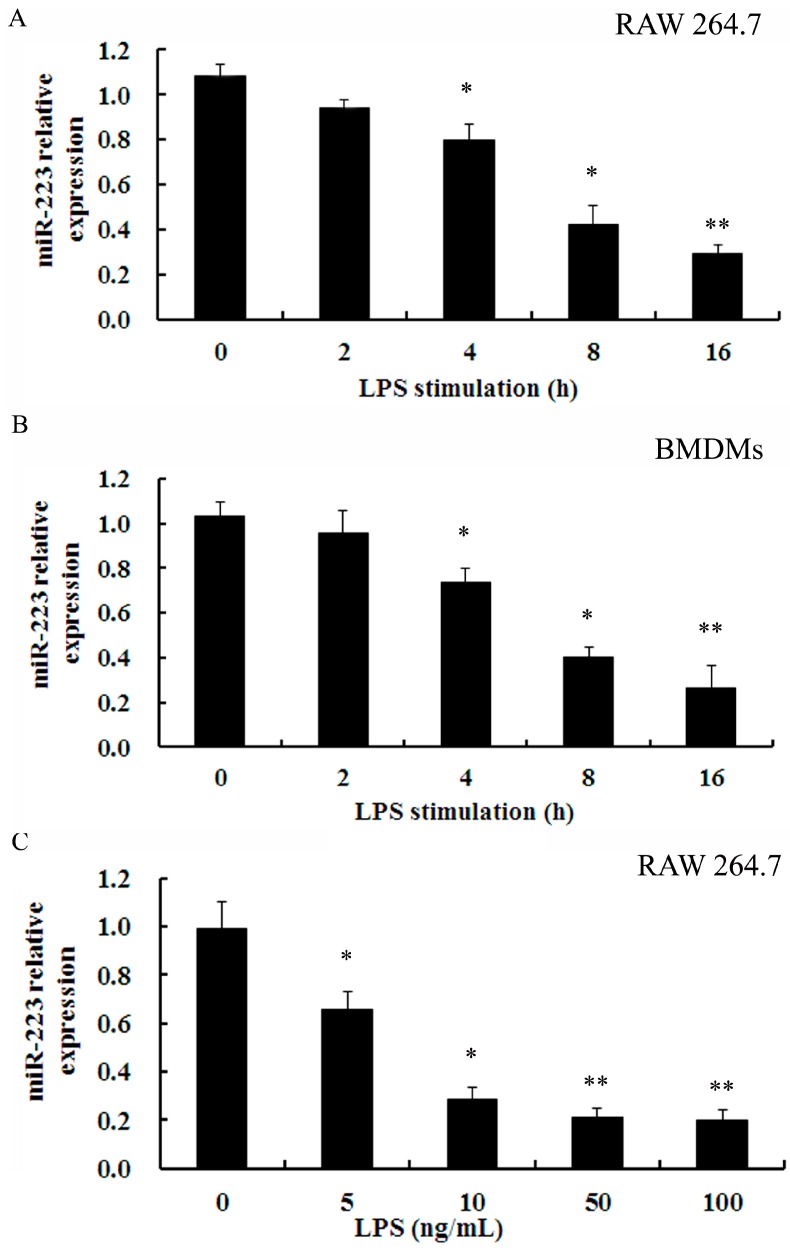
LPS inhibited the expression of miR-223 in RAW 264.7 and BMDMs. (**A**,**B**) RAW 264.7 cells and BMDMs were treated with 50 ng/mL LPS for the various times (0, 2, 4, 8 and 16 h). Then, the qRT-PCR was performed to detect the expression of miR-223; and (**C**) Following stimulation with the indicated doses of LPS for 16 h, the expression of miR-223 was assessed. * *p* < 0.05, ** *p* < 0.01 *vs.* control group. BMDMs: bone marrow-derived macrophages; LPS: lipopolysaccharide.

Macrophages are the principal effecter cells during the progress of atherosclerotic plaque formation and subsequent rupture. Hence, the expression of miR-223 in inflammatory macrophages triggered by LPS was investigated. As shown in Figure 2A, LPS induced a slight decrease in miR-223 levels at 2 h post-stimulation and an obvious down-regulation of miR-223 mRNA was observed during the next 2 h. Notably, the mRNA levels of miR-223 were gradually down-regulated with the inchmeal increased times of LPS stimulation in RAW 264.7 cells. Importantly, a similar down-regulation pattern for miR-223 expression was also confirmed in primary bone marrow-derived macrophages (BMDMs) (Figure 2B). Furthermore, the mRNA levels of miR-223 were about 0.28-fold and 0.21-fold over control when exposure to 5 and 10 ng/mL LPS, respectively (Figure 2C). Together, these results suggested a time- and dose-dependent decrease in miR-223 expression in LPS-triggered macrophages.

### 2.3. miR-223 Attenuated LPS-Triggered Foam Cell Formation

**Figure 3:**
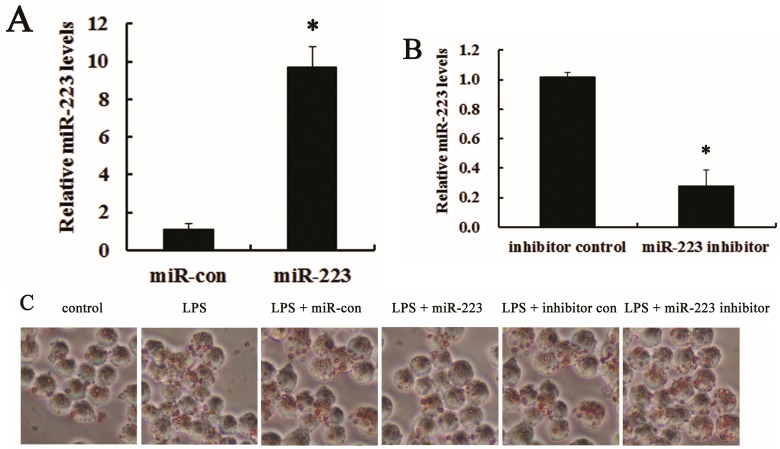

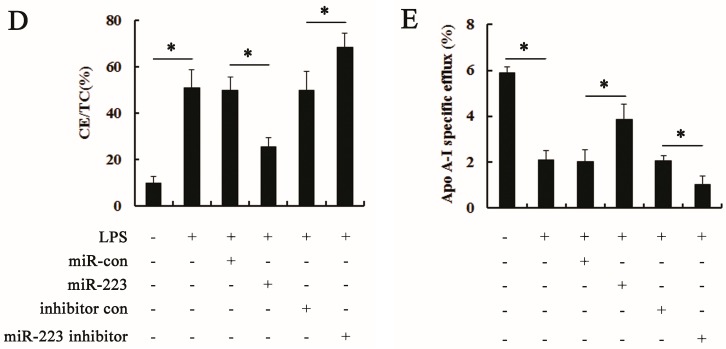
miR-223 reduced LPS-triggered macrophage foam cell formation. Following transfection with miR-223 mimics (**A**) or anti-miR-223 inhibitor (**B**), the expression levels of miR-223 in RAW 264.7 cells were assessed by qRT-PCR. RAW 264.7 cells transfected with miR-control or inhibitor control were used as control, respectively; (**C**) RAW 264.7 cells pre-treated with miR-223 mimics or inhibitors were stimulated with LPS (50 ng/mL, for 16 h), prior to incubation with ox-LDL (50 μg/mL) for 48 h. Oil red O staining was performed to analyze the effect of miR-223 on foam cell formation; (**D**) The ratio of cholesteryl ester/total cholesterol (CE/TC) was determined by HPLC to evaluate lipid deposition in LPS-triggered macrophages; and (**E**) After labeled with ^3^H-cholesterol (5 μCi/mL), the effect of miR-223 on cholesterol efflux from RAW 264.7 was investigated. The lower halves of Figure 3D,E are identical. * *p* < 0.05.

Macrophages have been widely accepted as critical for lipid-foam cell formation, a hallmark for atherosclerosis [35]. To further explore the role of miR-223 during atherosclerosis, we investigated its function in macrophage-derived foam cell formation triggered by LPS. Following transfection with miR-223 mimics, an obvious up-regulation of miR-223 mRNA was demonstrated in RAW 264.7 cells, compared with the control group (Figure 3A). Moreover, the anti-miR-223 inhibitor treatment dramatically reduced the expression levels of miR-223 mRNA (Figure 3B). Further analysis validated that miR-223 overexpression strikingly antagonized LPS-induced macrophage-derived foam cell formation, but increased it in miR-223 inhibitor-treated groups (Figure 3C). Moreover, elevation of miR-223 in macrophages significantly attenuated LPS-induced lipid deposition evidence as the ratio of cholesteryl ester/total cholesterol (CE/TC) decreased from 50.88% to 25.34%, but increased to 68.23% in miR-223 silencing groups (Figure 3D). Simultaneously, miR-223 up-regulation obviously accelerated cholesterol efflux to apoA-I (Figure 3E). A corresponding decrease in cholesterol efflux was observed when silencing miR-223 expression. Accordingly, the above data corroborated miR-223 might act as a negative regulator for macrophage foam cell formation.

### 2.4. miR-223 Negatively Regulated Inflammatory Response to LPS in TLR-Triggered Macrophages

**Figure 4:**
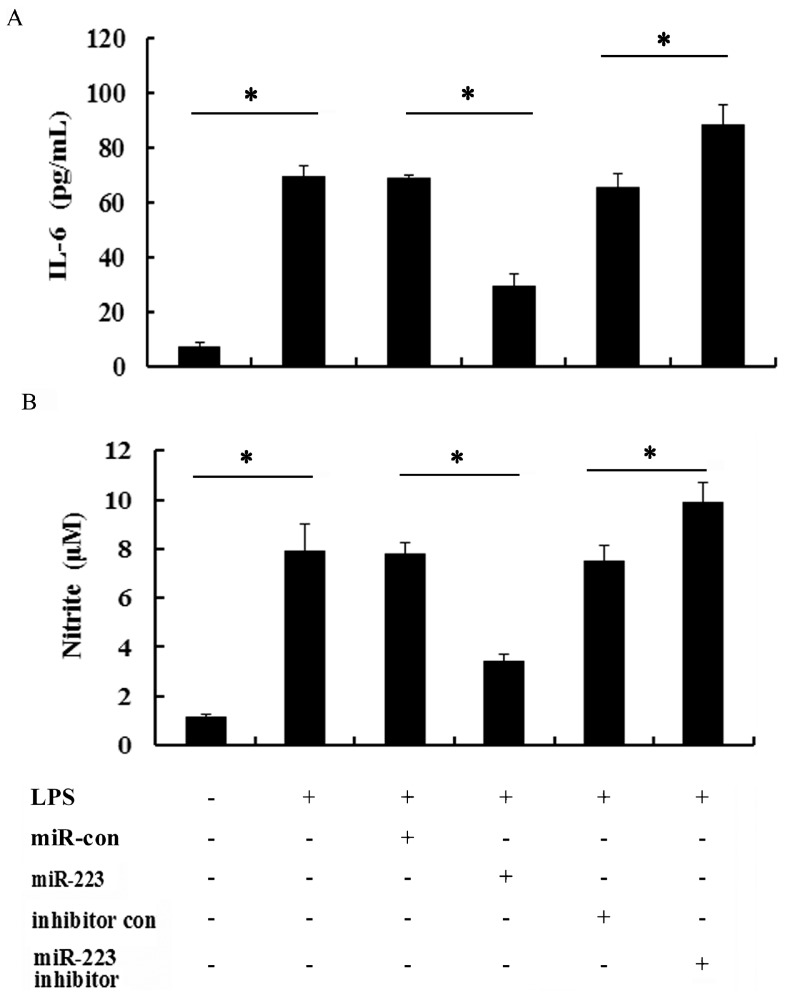
Effect of miR-223 on the production of inflammatory cytokines in LPS-activated macrophages. After transfection with miR-223 mimics or anti-miR-223 inhibitor, cells were exposed to LPS (50 ng/mL) for 16 h. Then, the levels of IL-6 were demonstrated by ELISA assay (**A**); the culture supernatants were subsequently isolated and analyzed for nitrite levels (**B**). * *p* < 0.05.

It is widely accepted that macrophage-mediated inflammatory response is critical for the development of atherosclerosis and plaque formation [36]. To further evaluate the effects of miR-223 on LPS-induced inflammatory response in macrophages, the pro-inflammatory cytokines of IL-6 and NO were determined. As shown in Figure 4A, stimulation of macrophages by LPS resulted in the significant production of IL-6, which was obviously down-regulated by the transfection of miR-223 mimics in activated macrophage. However, silencing miR-223 levels significantly augmented LPS-induced IL-6 production. Furthermore, miR-223 elevation remarkably attenuated nitrite production in RAW 264.7 macrophages exposure to LPS, but increased it in the anti-miR-223 inhibitor-treated groups (Figure 4B). These results indicated that miR-223 antagonized LPS-induced IL-6 and NO production, indicating its potential as a negative regulator to inflammatory response in TLR-triggered macrophages.

### 2.5. Activation of the TLR4-NF-κB Pathway Is Abrogated by miR-223 Mimics and Responsible for the Inhibitory Effect of miR-223 on Lipid Accumulation and Inflammatory Response

**Figure 5:**
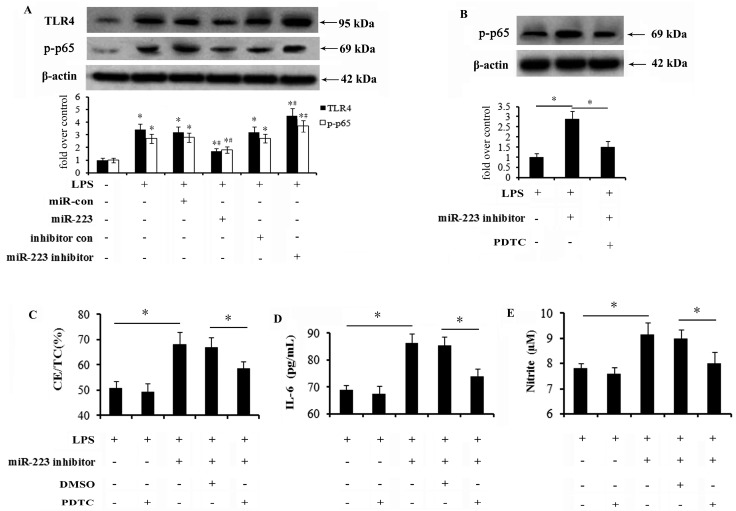
miR-223 antagonized lipid accumulation and inflammatory response in activated macrophages by suppressing TLR4-NF-κB pathway. (**A**) 48h after up-regulation or down-regulation of miR-223 levels, the activation of TLR4 and intra-nuclear NF-κB p65 was analyzed by western blotting. * *p* < 0.05, compared to the untreated control group. ^#^ *p* < 0.05, compared to the LPS treated control group; (**B**) Cells were treated with 30 μM NF-κB inhibitor PDTC for 4 h, prefer to exposure to LPS for 16 h. The effect of miR-223 silencing on the phosphor-p65 levels was confirmed by western blotting. * *p* < 0.05. The corresponding functions of PDTC pre-treatment on miR-223 down-regulation-triggered lipid accumulation (**C**), IL-6 (**D**) and NO (**E**) levels were also detected. The lower halves of Figure 5D,E are identical. * *p* < 0.05. TLR4-NF-κB: toll-like receptor 4 (TLR4)-nuclear factor-κB; PDTC: pyrrolidinedithiocarbamate.

To clarify the underlying mechanisms involved in the inhibitory effects of miR-223 on lipid deposition and inflammatory response in macrophages, TLR4 signaling, a key pathway during atherosclerosis, was investigated. Following transfection with miR-223 mimics, the increases in TLR4 and phosphor-p65 expression levels triggered by LPS stimulation were notably mitigated (Figure 5A). miR-223 silencing enhanced LPS-induced expression of TLR4 and its downstream signaling effector NF-κB. To further address the association between miR-223 and the TLR4-NF-κB pathway during macrophage-regulated lipid uptake and inflammation, we blocked this pathway by addition of PDTC, a specific inhibitor for NF-κB pathway. As expected, pretreatment with PDTC significantly abrogated the increase in phosphor-p65 expression triggered by miR-223 down-regulation (Figure 5B). Further mechanism assays corroborated that miR-223 down-regulation-induced increase in lipid accumulation was strikingly inhibited when pretreated with PDTC (Figure 5C). Precondition with PDTC also resulted in the similar down-regulation of IL-6 (Figure 5D) and NO (Figure 5E) levels in anti-miR-223 inhibitor-treated cells. Therefore, the data suggests that miR-223 could regulate lipid deposition and the inflammation response in macrophages triggered by LPS via suppression of TLR-4-NF-κB signaling.

### 2.6. miR-223 Mitigates TLR4 Signaling Activation through the PI3K/AKT Pathway

**Figure 6:**
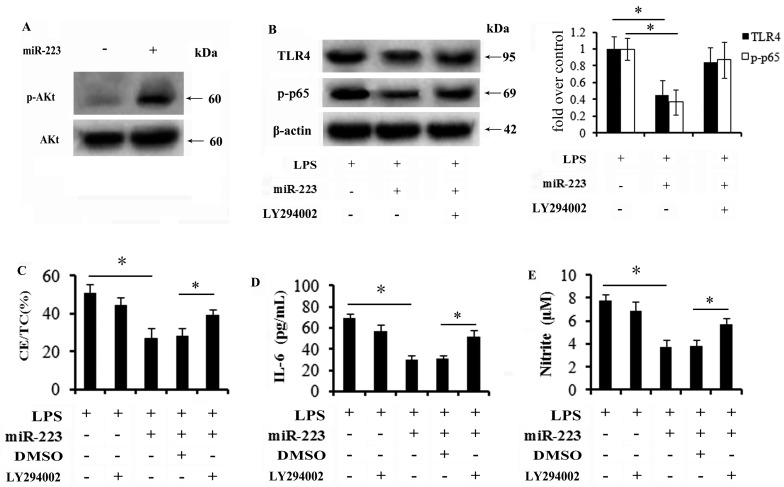
miR-223 mitigated TLR4 signaling activation primarily through the PI3K/AKT pathway. (**A**) Cells were transfected with miR-223 mimics. Forty-eight hours later, the activation of PI3K/AKT signaling was determined by western blot analysis; (**B**) Following pretreatment with 20 μM LY294002 (PI3K inhibitor) for 4 h, the effect of miR-223 on activation of TLR4-NF-κB pathway was analyzed. The subsequent effects on lipid deposition (**C**) and inflammatory cytokines levels (**D**,**E**) were further detected. * *p* < 0.05.

PI3K/AKT signaling has recently been recognized as a negative regulator for TLR4-mediated inflammation in several diseases [37]. To further investigate the underlying mechanisms involved in miR-223-regulated TLR-4-NF-κB signaling, activation of the PI3K/AKT pathway was investigated. Western blotting analysis conferred an obvious up-regulation of phospho-AKT (p-AKT) expression when cells were transfected with miR-223 mimics (Figure 6A). Importantly, miR-223 up-regulation significantly abrogated the activation of TLR4 and p65, which was notably increased when preconditioned with LY294002, a specific inhibitor for PI3K/AKT signaling (Figure 6B). Further mechanism analysis demonstrated that the inhibitor effect of miR-223 on lipid accumulation triggered by LPS stimulation was remarkably attenuated following LY294002 pretreatment (Figure 6C), concomitant with the corresponding increase in IL-6 (Figure 6D) and NO production (Figure 6E). Together, these results confirmed that miR-223 might block TLR4 signaling-triggered lipid accumulation and inflammation predominantly by activating the PI3K/AKT pathway.

## 3. Discussion

MicroRNAs (miRNAs) are highly conserved, small non-coding RNAs, and elicit crucial roles in multiple physiological and pathological processes by regulating target gene expression. Emerging evidence has confirmed the prominent function of miRNAs in cardiovascular disease [16,17,18]. Moreover, a selective microRNA-based strategy has been shown to inhibit restenosis while preserving endothelial function, indicating a critical role in the treatment of coronary artery disease (CAD) [27]. Atherosclerosis is an inflammatory-associated disease that is the major contributor for the development of cardiovascular disease [38]. MicroRNA-19b/221/222 [39], miR-21 [40] as well as miR-146a [41] were found to be involved in the process of atherosclerosis. miR-223 has recently been confirmed to be involved in several inflammatory diseases, including inflammatory bowel disease and type 2 diabetes [26,27]. However, its roles in atherosclerosis is still unclear. In the present study, we observed a significant increase of miR-223 in atherosclerotic plaque. Importantly, miR-223 synchronously attenuated lipid accumulation and inflammatory in activated macrophages by LPS stimulation through negatively regulating TLR4-dependent signaling pathway.

Atherosclerosis is recently understood as a chronic inflammatory vascular disease driven by the accumulation of macrophages. Increasing laboratory studies have indicated that macrophages are of central importance for the initiation and progression of atherosclerosis and that they have pivotal functions in inflammation and lipid deposition, both the key triggers for the formation and rupture of plaques [36,42]. Therefore, we investigated the levels and function of miR-223 on macrophage-induced foam cell formation and inflammation. In the current research, LPS dose- and time-dependently blocked the expression of miR-223 mRNA in RAW 264.7 and bone marrow-derived macrophages. However, an increased expression of miR-223 was observed in atherosclerotic lesions. The increased expression of miR-223 in atherosclerotic lesions might indicate a feedback mechanism. Following transfection with miR-223 mimics or anti-miR-223 inhibitor, a detailed study to investigate the involvement of miR-223 in macrophage foam cell formation was performed. Here, miR-223 overexpression obviously abrogated the ratio of CE/TC and subsequent lipid-loaded macrophage foam cell formation, concomitant with the increase of cholesterol efflux to apoA-I. Importantly, macrophages activated by LPS stimulation exhibited the increases in the production of pro-inflammatory cytokines IL-6 and NO, which were dramatically reduced by miR-223 transfection. Simultaneously, blocking miR-223 expression accelerated lipid deposition and inflammatory cytokine production in macrophages stimulated with LPS. These findings may suggest a new mechanism for the effect of miR-223 in atherosclerosis, potentially facilitating plaque stabilization.

The current notion that Toll-like receptor 4 (TLR4) plays pivotal functions during atherosclerosis has garnered increased interest. TLR4 is over-expressed in ruptured plaques in patients with acute myocardial infarction [43]. Once activated by LPS stimulation, a common receptor for TLR4, abundant inflammatory cytokines will be released from macrophages by activating its downstream NF-κB signaling in a myeloid differentiation factor 88 (MyD88)-dependent manner [44,45]. Moreover, the enhanced cholesterol loading of macrophage foam cells has been observed [11,12]. Importantly, injection with TLR4 ligand LPS accelerates the development of atherosclerosis and the formation of vulnerable plaque [46]. To further clarify the mechanism involved in the inhibitor effect of miR-223 on lipid deposition and inflammatory in macrophages, the TLR4 signaling was therefore explored. In accordance with our hypothesis, miR-223 up-regulation remarkably dampened the activation of TLR4 and its downstream NF-κB, but reversely increased the activation of TLR4-NF-κB by blocking miR-223 expression. Interestingly, the augmented effect of miR-223 silencing on lipid accumulation and inflammation in activated macrophages was obviously attenuated when preconditioned with PDTC. Accordingly, miR-223 could reversely regulate macrophage-mediated lipid deposition and inflammatory response primarily by suppressing TLR4-NF-κB signaling.

An increasing body of evidence has suggested that TLR4 represents a novel therapeutic target due to its vital role in plaque formation and instability. When silencing TLR4 levels in ApoE^−/−^ mice fed with HFD, the obvious decrease in vulnerable plaques resulted, and was accompanied with reduced lipid deposition and inflammatory cytokines levels [11,13]. Therefore, it is urgent to identify new possibilities to suppress TLR4 signaling during atherosclerosis. PI3K/AKT signaling is known to be involved in various disease pathological processes, such as hypertension [33]. Recently, the PI3K/AKT pathway ranks as a negative regulator of inflammatory response in several diseases [47,48]. During mouse liver ischemia/reperfusion injury, phosphatase and tensin homolog deleted on chromosome ten (PTEN) deficiency enhanced the activation of AKT signaling, which obviously inhibited the TLR4-mediated inflammatory pathway and ultimately attenuated inflammation injury in ischemia/reperfusion -stressed liver [48]. Furthermore, the activation of PI3K/AKT signaling also exerts anti-inflammatory effects on skeletal muscle cells through negative regulation of the TLR4 pathway [49]. To further elucidate the underlying mechanism involved in the inhibitor effect of miR-223 on TLR4 signaling during lipid uptake and inflammation in macrophages, we investigated Akt activity as well. As expected, miR-223 induced the activation of PI3K/AKT signaling. When blocking this pathway with the specific inhibitor LY294002, the inhibitory effect of miR-223 on the activation of TLR4-NF-κB signaling was attenuated. Simultaneously, the similar increase in inflammatory cytokines IL-6 and NO levels was also corroborated, indicating that PI3K/AKT may contribute for the inhibitor role of miR-223 on TLR4-mediated lipid accumulation and inflammation in macrophages. However, how does miR-223 exerts its regulatory role in the PI3K/AKT pathway? Are other signal mechanisms also involved in this process? Both of these questions will be explored in our future studies.

## 4. Experimental Section

### 4.1. Reagents and Antibodies

LPS from *Escherichia coli* (serotype 055:B5) was purchased from Sigma-Aldrich (Taufkirchen, Germany). The PI3K inhibitor LY294002 and NF-κB inhibitor PDTC were obtained from Calbiochem (San Diego, CA, USA). The primary antibodies against phospho-Akt (S473, rabbit polyclonal antibody) and total Akt antibodies (goat polyclonal antibody) were obtained from Cell Signaling Technology (Beverly, MA, USA). Rabbit anti-mice TLR4 polyclonal antibody was from Abcam (Cambridge, UK). The polyclonal antibody against phosphor-p65 and the peroxidase-conjugated secondary antibodies were purchased from Santa Cruz Biotechnology (Santa Cruz, CA, USA).

### 4.2. Animal Model of Atherosclerosis

Twenty apolipoprotein E (ApoE)^−/−^ mice (6–8 weeks old) were purchased from Changzhou Cavens Laboratory Animal Co., LTD (Changzhou, China) and randomly divided into two groups. All animals were housed under controlled 12-h light/dark cycle and temperature conditions, with free access to water and chow. To induce the development of atherosclerotic lesions, mice were fed with the high-fat diet (HFD, 20% fat, 20% sugar, and 1.25% cholesterol) for 3 months. We verified that this was successful by dosing cholesterol and triglycerides in the serum of the mice. C57BL/6J wild-type mice (Charles River Laboratories, Wilmington, MA, USA) served as controls. At the end of this period, the mice were sacrificed. The aorta specimens were collected and frozen in liquid nitrogen for RNA extraction. All the experiments were conducted under a protocol approved by the Institutional Animal Care and Use Committee of the First Affiliated Hospital of Medical College, Xi’an Jiaotong University. All efforts were made to minimize suffering.

### 4.3. Macrophage Cell Culture and Treatment

Murine macrophage cell line RAW 264.7 was obtained from the American Type Culture Collection (ATCC, Manassas, VA, USA). Mouse bone marrow-derived macrophages (BMDMs) were isolated and cultured in MEM-α medium supplemented (Gibco, Grand Island, NY, USA) with 10% fetal bovine serum (FBS) (Gibco), 50 units/mL penicillin (Gibco), and 50 μg/mL streptomycin (Gibco) as previously described [50]. RAW 264.7 cells were cultured in DMEM (Dulbecco’s modified Eagle’s medium) (Gibco), including 2 mM l-glutamine (Sigma, St. Louis, MO, USA), 100 U/mL penicillin, 100 μg/mL streptomycin and 10% FBS. All cells were cultured at 37 °C under 5% CO_2_. After washed twice with fresh medium, cells were stimulated with the indicated dose and times of LPS, prior to incubation with ox-LDL (50 μg/mL) for 48 h.

### 4.4. Oligonucleotide Transfection

RAW 264.7 cells were seeded into 6-well plates. To specifically induce or silence miR-223 expression in macrophages, the miR-223 mimics and miR-controls, miR-223 inhibitor and inhibitor controls (10 nM) were all from GenePharma (Shanghai, China). For transfection, the above oligonucleotides were performed using Lipofectamine 2000 reagent (Invitrogen, Carlsbad, CA, USA) according to the manufacturer’s protocol. After transfection for 48 h, cells were harvested for further experiments and the transfected efficiency were evaluated using quantitative RT-PCR.

### 4.5. RNA Extraction and Quantitative RT-PCR

Total RNA from aorta and macrophages was isolated using TRIzol reagent (Life Technologies, Gaithersburg, MD, USA). About 5 μg total RNA from each sample was reverse transcribed into first strand cDNA with using the cDNA Synthesis Kit (Fermentas, St. Leon-Rot, Germany). Then, the acquired cDNA was conducted as a template to perform real-time PCR reactions using SYBR Premix Ex TaqTM II Kit (Takara, Dalian, China) according to the manufacturer’s instructions. About 10 μmol/L sepcific primer for miR-223 was used as previously described [51] and obtained from Takara. The expression level of miR-223 in each sample was normalized to U6 in the same sample according to 2^−ΔΔ*C*t^.

### 4.6. Oil Red O Staining

Following treatment with LPS for the indicated doses and times, the cultured macrophages were rinsed with PBS buffer for three times. Then, cells were fixed with 4% paraformaldehyde/PBS for 15 min, followed by the staining with the freshly diluted 0.5% Oil red O solution (Sigma) at 37 °C. About 10 min later, hematoxylin was further used to label cell nuclei. Foam cells were counted under a microscope, and the Oil red O staining was assessed by a color density assay using iVision software (BioVision Technologies, Exton, PA, USA).

### 4.7. Lipid Assay by High-Performance Liquid Chromatography (HPLC)

To analyze lipid deposition in macrophage-derived foam cells, cellular lipid contents were detected by HPLC, including total cholesterol (TC) and cholesterol ester (CE). Following exposure to ox-LDL, 0.9% NaOH solution was used to lyse cells and the total protein concentration was detected by BCA kit. Then, an equal volume of trichloroacetic acid was added. After centrifugation at 800× *g* for 10 min, the residues were re-suspended in 100 μL of isopropanol-acetonitrile (*v*/*v*, 20:80). All the samples were performed by Agilent 1100 series HPLC (Wilmington, DE, USA). Lipid accumulation levels were shown as the ratio of CE/TC.

### 4.8. Cholesterol Efflux Analysis

The above cells were pre-treated with ^3^H-cholesterol (5 μCi/mL) for 12 h in basic medium. After equilibration of base medium for 2 h, cholesterol efflux was then stimulated by incubation with 10 μg/mL apoA-I for 8 h. All the specimens were subjected to FJ-2107P type liquid scintillator to ascertain the radioactivity. Cholesterol efflux was expressed as the percentage of radioactivity released into the medium relative to total radioactivity in cells plus media.

### 4.9. Measurement of IL-6 Cytokine Production

The interleukin 6 (IL-6) levels in the above macrophages were performed using Enzyme-linked immunosorbent assay (ELISA) assay. Briefly, about 2 × 10^5^ cells were seeded into 24-well plates. Following stimulated with 100 ng/mL LPS for 12 h, IL-6 concentration from cell-culture supernatants were measured by IL-6 ELISA kit (BioSource International, Camarillo, CA, USA) using ELISA DuoSet Development systems according to the manufacturer’s instructions.

### 4.10. Determination of NO Concentration

The collected culture supernatant was added into 100 μL Griess reagent (mixture of equal volume of 1% sulfanilamide in 5% H_3_PO_4_ and 0.1% *N*-(1-naphthyl) ethylenediamine dihydrochloride in H_2_O) in a 96-well plate. Then, the spectrophotometric measurement was performed at 550 nm. The nitrite concentrations in the supernatants, a stable oxidized product of NO, were determined by comparison with a sodium nitrite standard curve.

### 4.11. Western Blotting Assay

RAW 246.7 cells were homogenized and lysed with RIPA lysis buffer (100 mM NaCl, 50 mM Tris–HCl pH 7.5, 1% TritonX-100, 1 mM EDTA, 10 mM b-glycerophosphate, 2 mM sodium vanadate and protease inhibitor). The total protein concentration was assessed with the micro-BCA protein assay (Pierce, Rockford, IL, USA). Following separated by 12% polyacrylamide gel, the protein was electroblotted onto the polyvinylidene difluoride (PVDF) membrane. The nonspecific binding was blocked by incubating with 5% nonfat milk in Tris-buffered saline with Tween (TBST) buffer at room temperature for 1 h. Then, the membrane was incubated with the primary antibodies against TLR4, phosphor-p65, p-AKT, and AKT for 1 h. After washing with TBST, HRP-conjugated second antibodies were added for 1 h. Then, the bound antibodies were visualized using LumiGLo reagent (KPL, Gaithersburg, MD, USA).

### 4.12. Statistical Analysis

Data are presented as mean ± SEM. All statistical analyses were performed using SPSS 11.0 software (SPSS Inc., Chicago, IL, USA). Data comparison between two groups was performed using a Student’s *t* test. For multiple sets of data analysis, the ANOVA assay was used. Statistical significance was considered significant when *p* < 0.05.

## 5. Conclusions

In conclusion, this research demonstrates the expression of miR-223 in plaques and down-regulation in activated macrophages with LPS stimulation. Importantly, we demonstrate that miR-223 might negatively regulate TLR4 signaling-triggered lipid accumulation and inflammation primarily by activating the PI3K/AKT pathway in macrophages. Therefore, miR-223 may be a promising therapeutic agent against cardio-cerebral vascular disease. However, this study only demonstrated the mechanisms involved in anti-lipid deposition and the inflammatory response effect of miR-223 predominantly by negatively regulating TLR4 signaling *in vitro*, but did not to provide direct evidence of its anti-atherosclerosis function by regulating lipid metabolism and inflammation through the TLR4 pathway *in vivo*. Further research will focus on its effect on lipid levels and inflammation in atherosclerotic plaques *in vivo*. Moreover, the TLR4 signaling involved in this process will also be investigated in animal models of atherosclerotic mice.
